# The effect of environmental variations on the production of the principal agricultural products in Colombia

**DOI:** 10.1371/journal.pone.0304035

**Published:** 2024-07-05

**Authors:** Carlos Felipe Cortés-Cataño, Yennifer Foronda-Tobón, Jairo Armando Paez-Ricardo, Jairo Enrique Parra-Herrera, Mario Julian Cañon-Ayala

**Affiliations:** 1 School of Information and Communication Technologies, Politécnico Grancolombiano University, Bogotá, Colombia; 2 School of Optimization, Infrastructure and Automation, Politécnico Grancolombiano University, Bogotá, Colombia; Balochistan University of Information Technology Engineering and Management Sciences, PAKISTAN

## Abstract

The agricultural sector of Colombia supports the national economy and food security due to the rich lands for cultivation. Although Colombia has a vast hydrological basin, climate change can impact agricultural productivity, generating economic and social adverse effects. For this, we evaluated the impact of some environmental variables on the production of the most sold crops using production, climatic, and hydrological data of the 1121 municipalities from 2007 to 2020. We modeled the production of coffee, rice, palm oil, sugarcane, and corn, adopting a Bayesian spatio-temporal model that involved a set of environmental variables: average temperature, minimum temperature, maximum temperature, evapotranspiration, precipitation, runoff, soil moisture, vapor pressure, radiation, and wind speed. We found that increases in the average temperatures can affect coffee (-0.2% per °*C*), rice (-3.76% per °*C*), and sugarcane (-0.19% per °*C*) production, meanwhile, these increases can boost palm oil (+2.55% per °*C*) and corn (+1.28% per °*C*) production in Colombia. This statement implies that the agricultural sector needs to substitute land use, promoting the production of palm oil and corn. Although our results did not find a significant effect of hydrological variables in any crop, suggesting that the abundance of water in Colombia might balance the impact of these variables. The increases in vapor pressure impact all the crops negatively (between -11.2% to -0.43% per *kPa*), except rice, evidencing that dry air conditions affect agricultural production. Colombia must manage the production location of the traditional products and implement agro-industrial technologies to avoid the climate change impact on crops.

## Introduction

The agricultural sector of Colombia employs around 38% of the 111.452.998 *ha* due to the abundance of water supply in the tropical equatorial region [1]. This sector contributed to 8.4% of the national gross domestic product in 2020 with products such as coffee, oil palm, and rice [2]. Colombia has maintained stable growth in this sector since the middle of the XX century despite the development of other economic activities [3, 4]. This sector has a crucial role in addressing food security and the economic development of Colombia.

Colombia has ecosystems with climatic conditions that allow the production of high-quality commodities such as coffee, rice, corn, sugarcane, and palm oil. For instance, the Andean region of Colombia maintains temperatures between 18 to 21°*C* that allow coffee production in the optimum conditions [5]. Valle del Cauca had temperatures between 20 to 30°*C*, the optimal conditions for sugarcane growth with enough water resources [6]. This range of temperatures is also optimal for planting rice although rice production in Colombia typically occurs in higher temperatures in Tolima, Huila, and Llanos Orientales [7]. Corn is one of the principal products of Colombia because this plant can grow at altitudes ranging from 0m to 3000m above sea level [8]. The optimal temperature conditions for corn cultivation range between 19°*C* to 29°*C*, depending on the variety. Oil palm also obtains optimal production close to 27°*C* with enough humidity in hot conditions [9]. These environmental conditions evidence that Colombia has the potential to develop these crops due to its optimal planting conditions and sufficient water resources.

Nevertheless, climate change might impact agricultural production because the increase in temperatures upsets the environmental conditions and water supply, inducing risk to food security [10]. The Food and Agriculture Organization of the United Nations (FAO) estimated a fall in agricultural productivity due to variations in environmental conditions, implying the necessity of improving this sector [11, 12]. Climate change can impact the agricultural sector worldwide, and Colombia could face economic and social difficulties due to its dependence on this sector [13].

Weather changes might affect the crops in Colombia, generating losses for producers and affecting food security [14]. Products such as coffee, palm, corn, sugarcane, and rice are vulnerable to floods, droughts, and temperature increases. Coffee is sensitive to plant diseases due to temperature changes, and this condition also promotes pest development [15]. According to the World Bank, climate change will affect Colombian agriculture, requiring crop adaptation by 2050. The coffee will require higher altitudes and shade than the current production conditions. Sugarcane, rice, and corn production will require adaptation to high temperatures and less water consumption [16].

The National Planning Department of Colombia (DNP) has adopted a policy for adapting the agricultural sector using climatic data and forecast models [17]. Although previous works estimated the fall in production due to the decline in available land for cultivating products such as rice by 2050, the improvement in technologies and the adaptation to other crops might mitigate the impact of climatic change on the Colombian economy [15, 18, 19]. Previous works also evaluated the variation and management in water supply, evidencing that the droughts and rainy seasons have provoked hydrological stress impacting the agricultural yield in Colombia [20–22]. These efforts evidence the necessity of analyzing the production and the effect of environmental changes.

We developed a statistical analysis for evaluating the impact of environmental changes on agricultural production in Colombia, adopting a Bayesian model that implements crop production and satellite data. This structure can estimate the perceptual variation in crop production due to temperature, water, and soil variations. The results can reveal the impact of climate change on the main agricultural products of Colombia.

## Materials and methods

We developed a statistical model to measure the relationship between crop production and environmental conditions. We selected coffee, corn, palm oil, rice, and sugarcane because these crops obtained the highest production rate between 2007 and 2020. We also compare the yearly production of these crops to a set of environmental variables: average temperature, maximum and minimum temperature, precipitation, evapotranspiration, runoff, soil moisture, wind speed, vapor pressure, and radiation. We adopted a spatio-temporal Bayesian model to find the relationship between crop production and environmental variables. This model represents the yearly production per municipality using the environmental variables as co-variables. Each co-variable has a random coefficient that describes the impact on crop production.

### Data

The yearly crop production was obtained from the Colombian Agricultural and Rural Development Ministry through *Agronet*, a free-access website that accounts for crop production per municipality [23]. We obtained the yearly data in tons for coffee, corn, palm oil, rice, and sugarcane at the municipality level from 2007 to 2020. We assessed the geographic division codes of Colombia, municipality coordinates, and maps from the Colombian Administrative Department of Statistics [24]. We created a database with 78.470 records of crop production (5 products) per municipality (1121 municipalities) during 14 years (2007-2020). We obtained the environmental variables from the TerraClimate website, which provides a dataset of monthly climate variables for ecological and hydrological studies [25]. We developed an R code to obtain each variable for each municipality coordinate per month from January 1958 to December 2020. Then, we calculated the variables at the yearly level as Table 1 summarizes.

**Table 1:** Environmental variables.

Variable	Units
Minimum temperature *	°*C*
Maximum temperature *	°*C*
Average temperature	°*C*
Actual evapotranspiration ^+^	*mm*
Reference evapotranspiration ^+^	*mm*
Precipitation ^+^	*mm*
Runoff ^+^	*mm*
Soil moisture ^+^	*mm*
Vapor pressure ^+^	*kPa*
Downward surface shortwave radiation ^+^	*W*/*m*^2^
Wind speed ^+^	*m*/*s*

* yearly maximum or minimum values.

^+^ yearly average per month.

### Descriptive analyses for temperature and presipitation

We also evaluated the environmental changes in Colombia using the temperature and precipitation data from 1960 to 2020 through maps and time series for both variables. The maps illustrate the average annual temp (°*C*) and the average precipitation (*mm*/*month*) per municipality divided by 12 years, adopting the *rgdal* package in *R* [26]. On the other hand, the time series measure the variables from 1958 to 2020 to represent the variations over time. We implemented these time series adopting the *ggplot2* package in *R* using the *geom*_*s*_*mooth* function for illustrating the best-smoothed regression between four linear regression models [27].

### Bayesian model

We implemented a Bayesian model for representing the yearly crop production in the 1121 municipalities of Colombia from 2007 to 2020. *y*_*it*_ represents production per municipality *i* at year *t* labeling *i* as *i* = {1, 2, …1121}, and *t* as *t* = {1, 2, …, 14}. *y*_*it*_ was represented as Poisson counts with mean λ_*it*_:

$$\begin{array}{c} y_{it}∽Poisson\left(\lambda_{it}\right) \end{array}$$ (1)

where λ_*it*_ = *ρ*_*it*_*ϵ*_*it*_ contains the production rate *ρ*_*it*_ and the production from the previous year *ϵ*_*it*_ as offset. *ρ*_*it*_ was represented in a logarithmic scale adopting linear predictors of random effects:

$$\begin{array}{c} \eta_{it}=log\left(\rho_{it}\right)=\alpha +\gamma_{i}+\delta_{t}+\beta X_{it} \end{array}$$ (2)

This linear predictor considers *α* as the average production in all municipalities, *γ*_*i*_ as the municipality random effect according to independent Gaussian random effect (iid), *δ*_*t*_ as the yearly random effect according to Random Walk Model (RW1) [28], and *β* is the effect of the environmental variable *X*_*it*_. *β* represents the growth rate in production due to the increase in the environmental variable, and this coefficient reveals the impact of the environmental variable on crop production. We applied this model to each crop (5 crops) per variable (11 variables) to avoid estimation errors due to the dependence between environmental variables.

We adopted the Integrated Nested Laplace Approximation (INLA) for model estimation through the INLA *R* package [29, 30]. We assume the default prior distributions (*logGamma*(0, 0.00005)) to *γ*_*i*_ and *δ*_*t*_ effects in model estimation.

## Results

### Descriptive analyses for temperature and presipitation

**Fig 1:**
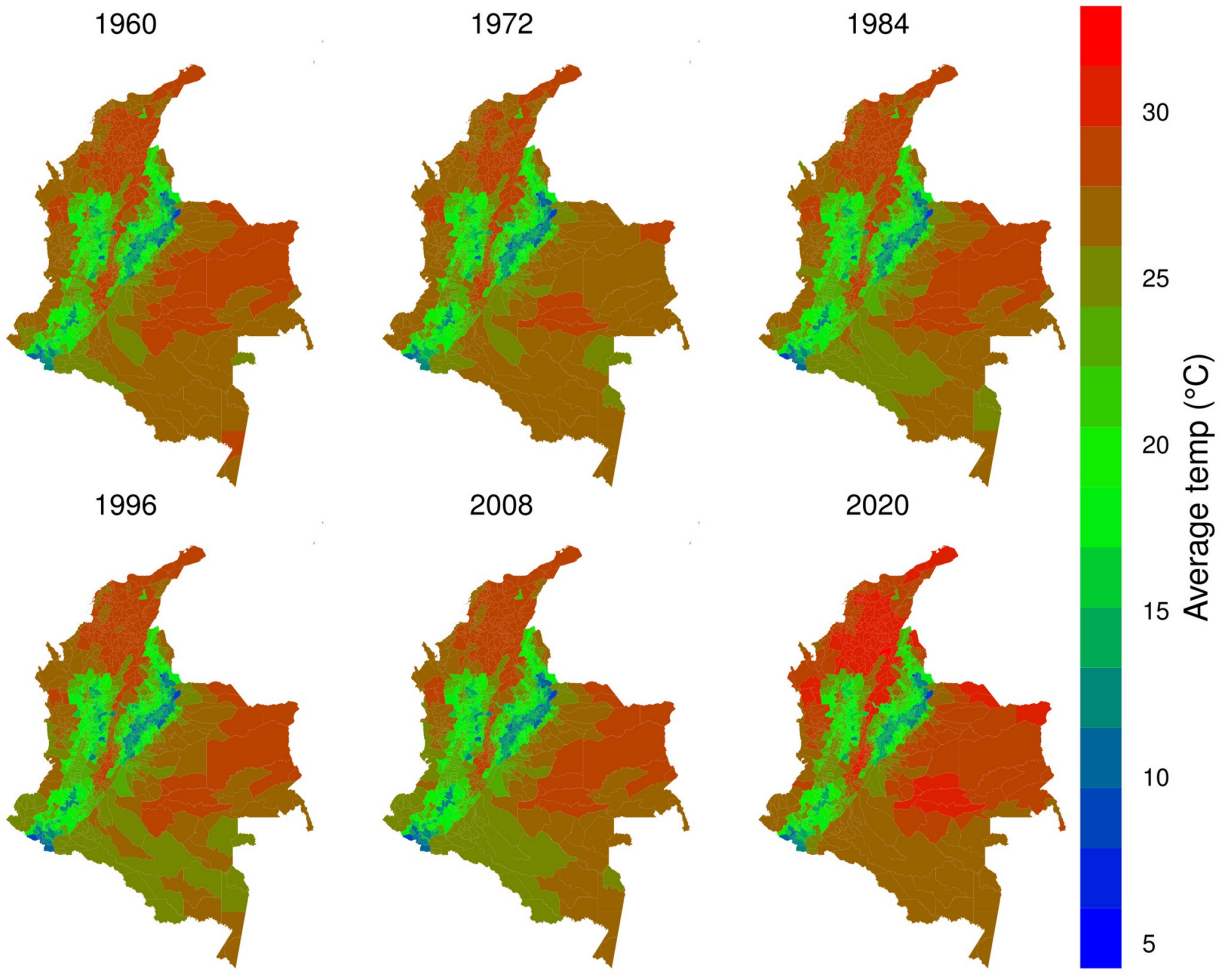
Average annual temp per municipality from 1960 to 2020.

The satellite data from Terraclimate evidence an increase in the average temperatures in Colombia, as Figs 1 and 3 illustrate. The central region of Colombia contains the Andean mountains, where the maps showed temperatures between 5°*C* to 20°*C*, and the rest of Colombia corresponds to a vast flat territory in the east, south, and north regions with temperatures above 25°*C*. 2020 was the hottest year in 60 years, and the smoothed series indicates the rise in the average temperature trend since 1970. The XXI century has experienced increases at a higher rate, suggesting a climatic change process in Colombia.

**Fig 2:**
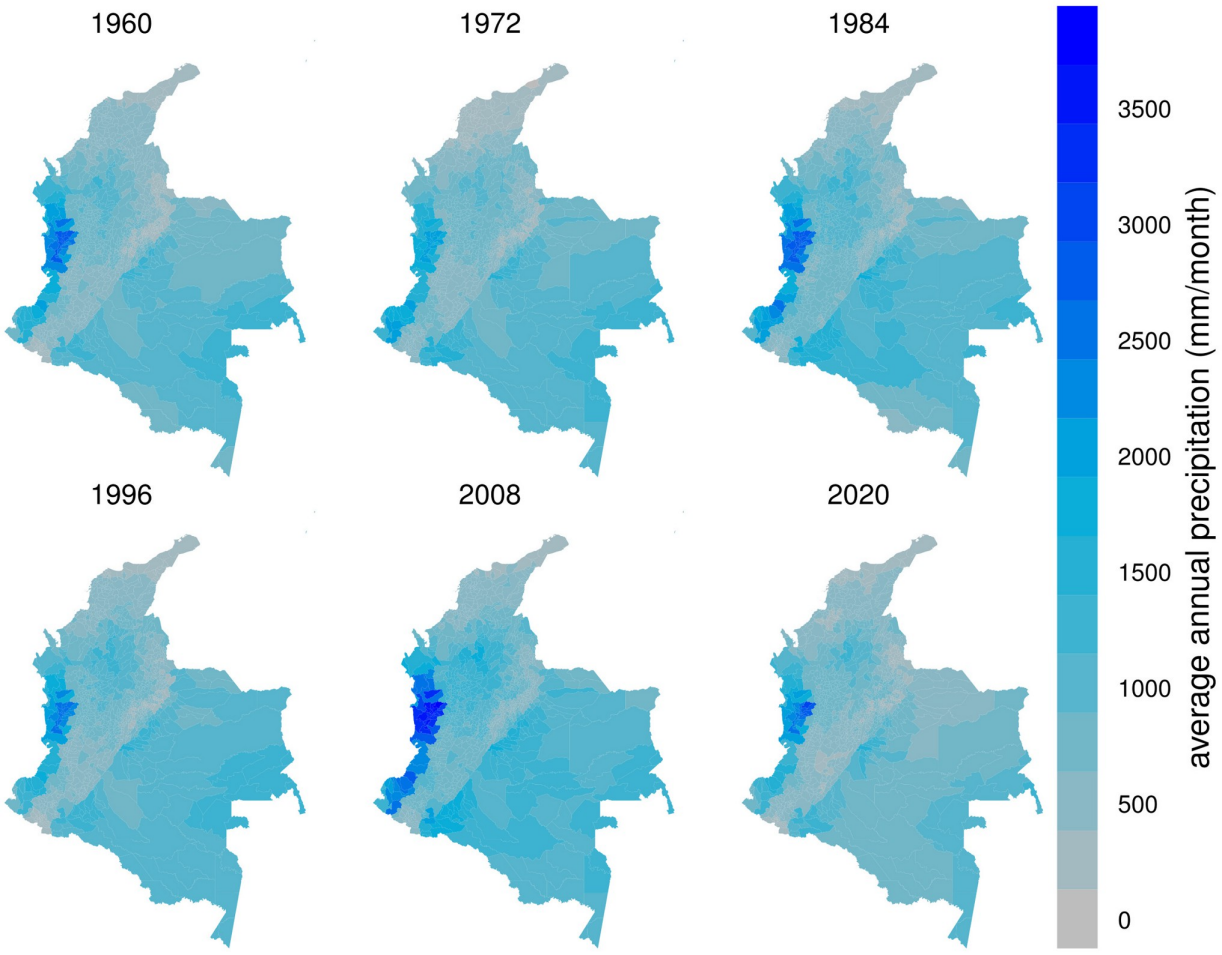
Average annual precipitation (mm/month) per municipality from 1960 to 2020.

**Fig 3:**
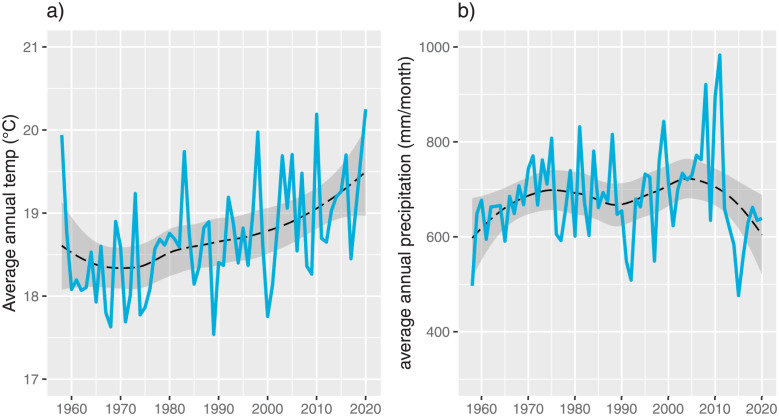
Average annual temp per municipality (Figure a) and average annual precipitation (mm/month) per municipality from 1960 to 2020 (Figure b).

The precipitation in Colombia has followed a random pattern comparing across years, in contrast to the temperature increase (see Fig 2). The western region of Colombia has been the wettest region, with precipitation above 3000 mm per month, while the north has experienced the least precipitation. Although the Andean region in the center of Colombia has a greater variety of crops, this region has the lowest precipitation levels than the east and south regions. The XXI century has experienced higher variability in the annual precipitation, showing the wettest year in 2011 and the driest year in 2015 (see Fig 3).

### Descriptive analyses of crop production

**Fig 4:**
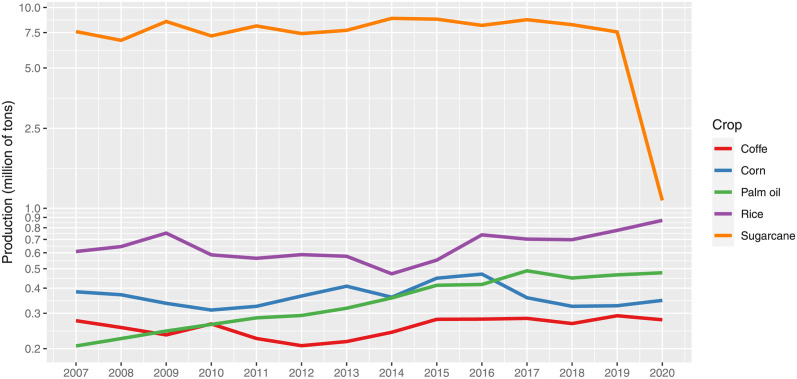
Major agricultural products in Colombia (annual production).

Sugarcane production was highest from 2007 to 2020, despite a drop in production in 2020 (see Fig 4). Rice has maintained second place in Colombia, and this crop has increased production since 2014. Coffee has followed a similar trend since 2015, and corn experienced the least productive period from 2017 to 2020. Palm oil production has experienced a constant rise, surpassing coffee and corn production.

**Fig 5:**
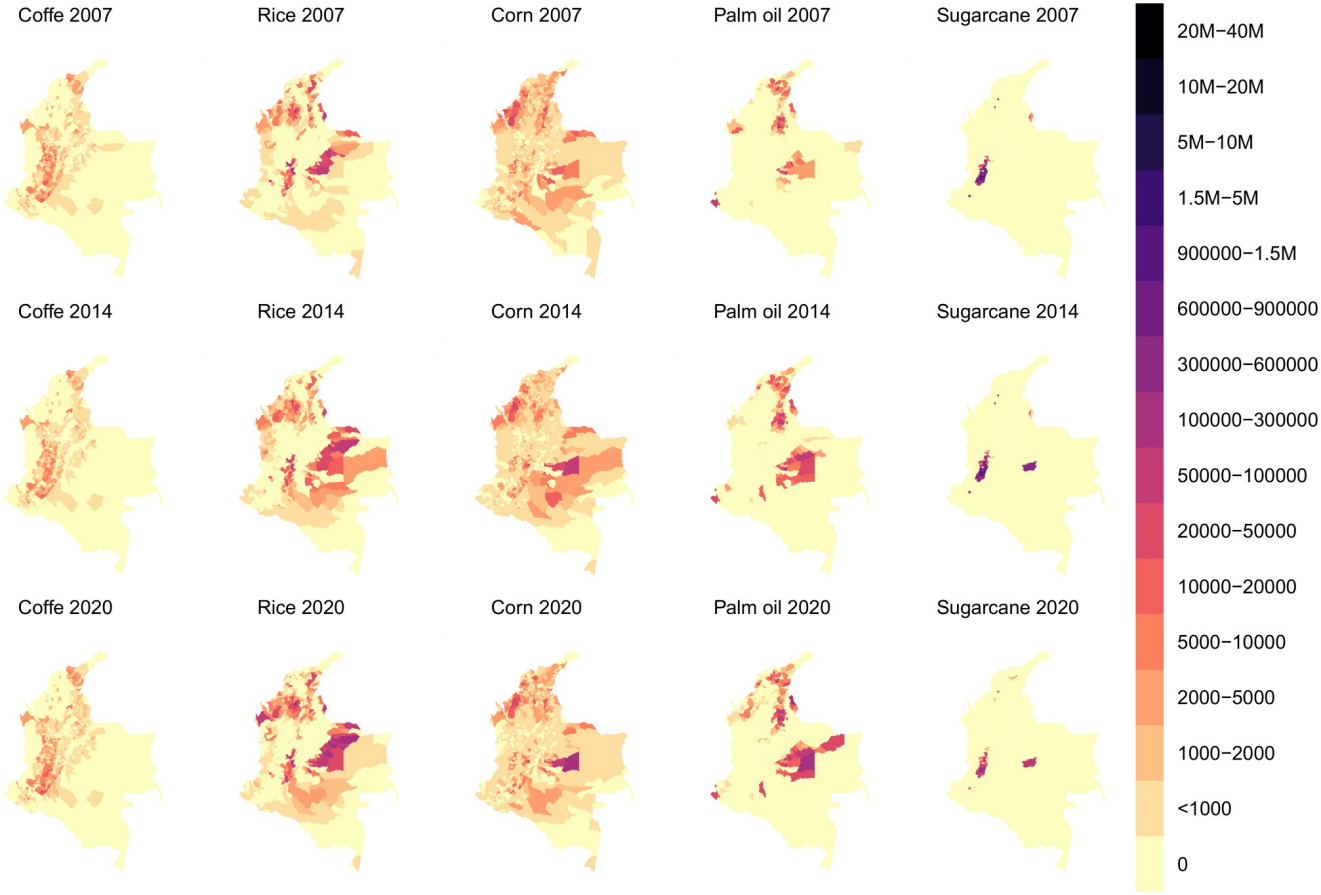
Main crops in Colombia per municipality in 2007, 2014, and 2020.

Although sugarcane production was the highest, only a few municipalities produced sugarcane, as Fig 5 illustrates. Northern and western regions of Colombia presented rice production clusters because these regions are in low-altitude areas. The Andean region had the primary lands for producing coffee, and the spatial distribution of municipalities indicated a similar trend between periods. The production map evidenced corn production in several areas across all regions of Colombia due to the capacity of this product for growing in different weather conditions (see Fig 5). Colombia has shown a land increase in producing palm oil throughout this period.

### Bayesian model

**Fig 6:**
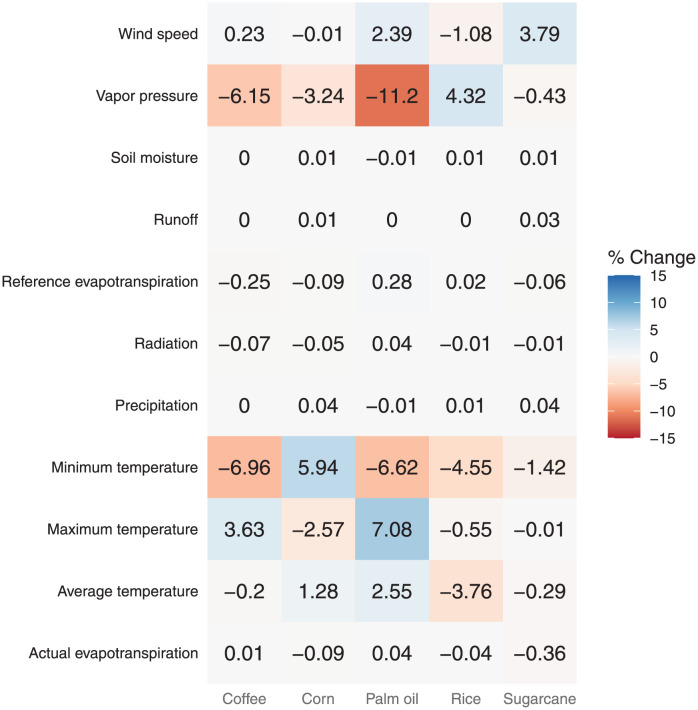
Mean-perceptual production increases due to the rise by a unit in the environmental variable.

We calculated the growth rate in production due to the environmental variable *β*, evaluating the exponential of the logarithmic scale value. These values represent the average increase in production perceived due to the rise by one unit in the environmental variable. The vapor pressure and the minimum temperature were the most impactful variables on crop production (see Fig 6).

Whereas the average temperature coefficients indicated that an increase in the average temperature might help corn and palm oil production, an increase in the average temperature might affect the production of coffee, sugarcane, and rice. Although the minimum temperature also showed a similar effect on crop production, increases in the minimum temperature could affect palm oil production. On the other hand, an increase in the maximum temperature only could help increasing coffee and palm oil production. Therefore, an average temperature increase could help increase corn and palm oil production, while increases in minimum and maximum temperatures might impact crop production at different levels.

Wind speed and vapor pressure indicated different effects between crops. The vapor pressure showed negative coefficients for coffee, corn, palm oil, and sugarcane production, suggesting that dry-air conditions might decrease their production. In contrast, dry air conditions might increase rice production. While an increase in wind speed might help palm oil and sugarcane production, this increase might impact corn and rice production. The coefficients of soil moisture, runoff, precipitation, radiation, and evapotranspiration variables showed a less significant impact than the other variables.

## Discussion

We found that increases in the average temperatures can affect coffee, rice, and sugarcane production, but meanwhile, these increases can boost palm oil and corn production in Colombia. This statement implies that the agricultural sector in the country needs to adjust land use, promoting the production of palm oil and corn. This result links to previous works that found that corn production could have positive results through the increase in temperature in cold and dry areas [31, 32]. Nevertheless, a previous study found that crop production yielded better results in temperate than in warm locations because the temperature increases affect plant cell development [33].

The current results evidence the advantages of promoting palm oil production within Colombia, and the recent increase in production reflects this as a priority in the national policy. This statement supports the National Federation of Palm Oil Growers (Fedepalma) projections for expanding domestic and international markets [34]. One previous work found that South America might maintain oil palm production instead of decreasing, as Southeast Asia expects [35]. This finding is consistent with the current research. Nevertheless, the increase in palm oil production might lead to deforestation, as the Southeast Asian region has experienced, resulting in adverse environmental and social impacts [36, 37].

Our results evidenced a positive effect of increasing average temperatures on palm oil production, but external factors such as fungus and viral diseases might negatively affect this crop. A previous study in Southeast Asian countries forecast an increase in the mortality rate of palm oil in climatic change conditions, suggesting the development of methods for mitigating the effect of climate change [38]. Fedepalma highlighted technologies and policy improvement for controlling pests and diseases in Colombia [39]. This implies the necessity of subsequent works to incorporate variables related to plant mortality to assess the impact of pests and diseases on crop production, as the current results only considered environmental variables.

Colombia has diverse weather conditions per region, maintaining similar conditions all year due to its location in the tropical zone. These weather conditions are competitive advantages for producing agricultural products such as coffee, sugarcane, and palm oil. However, some weather factors harmed crop production between 2007 and 2020. Factors related to droughts, such as the maximum and minimum temperatures, and the wind speed, might lead to crop failures in Colombia as Fig 6 illustrates. Previous work also evidenced crop failures related to droughts on the Asian coast in that period [40]. Nevertheless, the vast hydrological resources of Colombia and the resilience of Colombian farmers have helped to deal with the drought season, linking with our results that evidenced a low impact of hydrological variables on crop production [41].

Coffee represents the main commodity in Colombia, and the results evidenced a negative effect on its production due to the increase in the minimum temperatures and vapor pressure. Colombia has coffee crops below 1000*m* in altitude, and previous studies evidenced that higher temperatures affect coffee crops in low-altitude, with higher vapor pressure, linking with current results [42, 43]. On the other hand, the effect of higher temperatures is lower in high altitudes, according to Bunn et al., and this effect might explain the positive coefficient of the maximum temperature in the current study [42]. A previous study also evidenced divergences in the consequences of the temperature increase on coffee production, showing that temperature variations between 1.4°*C* and 3.1°*C* decreased the production by 11%. Meanwhile, this variation increased production in municipalities located in high-altitude areas by 2.3% [44]. Our results evidenced decreases in production due to the variations of minimum temperatures and vapor pressure linking with previous works that inferred problems in plant growth and development in adverse climatic conditions [45, 46]. These results imply the substitution of coffee crops in low altitudes to higher altitudes and the need to develop agroindustrial strategies to avoid the impact of temperature increasement.

Colombia has a large rice industry that requires efforts in management and economic support due to the high internal demand. Our results showed that the temperature increase decreased rice production, illustrating the crop vulnerability in the climatic change scenario. In this way, the Financial Fund for Development Projects of Colombia (FONADE) directed a study that estimated an adverse effect of the temperature increase on the flowering stage, declining the grain development by 10% in Colombia [47]. Moreover, The drought season in Colombia called “El Niño” decreases rice production, as previous work evidenced [48]. Ferreira et al. found that rice requires higher temperatures, increasing productivity, and this happens in low-altitude areas linked to the positive vapor-pressure coefficient that we obtained [32]. Although our results stand out a lower effect due to precipitation, runoff, and soil moisture, the rice requires vast amounts of water, and the rainy season called “La Niña” could benefit this crop [49]. Global warming can decrease rice production, but there are some strategies for adapting to these conditions, as a previous study estimated a production increase in temperature and rainfall variation [50, 51].

Sugarcane was the Colombian crop with the highest production during the study period, despite the few municipalities that cultivate this product. Our results revealed a negative effect of temperature increase on sugarcane but at a lower level than rice and coffee. This crop adapts easily to hotter temperatures in areas with heavy precipitation, showing that sugarcane would maintain its production in a country such as Colombia [52]. This statement might explain the lower impact of environmental variables in our results. Colombia has improved the sugarcane production process through agro-industrial technologies, mitigating the climatic-change impact, and environmental-rainy events such as “La Niña” season also help to maintain the production of this crop [49, 53]. Our results support the maintenance of sugarcane production in Colombia because the environmental variables obtained a lower impact on this crop, agreeing with previous studies.

Current results include the effect of environmental variables on crop production, but these results have a limited scope in estimating the impact of pests, plant diseases, and soil fertility. The increases in average temperatures might change the natural equilibrium of the pathogens’ population, inducing the emergence and the dispersion of pests and diseases [54]. It is an underestimated effect of the current study. On the other hand, the agricultural activities in Colombia have contributed to soil degradation, and this is also an underestimated effect of the work, despite the lower impact of soil moisture in the model [55]. Future studies should extend the inclusion of variables related to plant diseases and soil nutrients at a national level. Nevertheless, our results confirm that traditional crops such as coffee and rice can reduce their production due to temperatures and vapor pressure increases, while the country is experiencing global warming [15].

Our results noted the necessity to adapt the agricultural sector of Colombia to promote products such as corn and palm oil, due to the negative impact of environmental changes on traditional products such as coffee and rice. Although our results did not find a significant effect of hydrological variables in any crop, subsequent studies have to consider the drought periods and the water availability that might balance the impact of these variables.

Colombia has experienced an increase in average temperatures, impacting the production of commodities such as coffee and rice. Colombia must manage the production location of the traditional products and implement agro-industrial technologies to reduce the climatic change impact on crops, as the sugarcane industry has done. Therefore, Colombia must develop programs for promoting crops with advantages in these conditions. Although the hydrological resources help to avoid the negative impact, extreme climatic conditions such as droughts and critical rainy seasons can unbalance the agricultural conditions, deserving the worthing for subsequent studies of environmental and biological factors on crop production.
