# The effect of environmental variations on the production of the principal agricultural products in Colombia

**DOI:** 10.1371/journal.pone.0348323

**Published:** 2026-04-30

**Authors:** Carlos Felipe Cortés-Cataño, Yennifer Foronda-Tobón, Jairo Armando Paez-Ricardo, Jairo Enrique Parra-Herrera, Mario Julian Cañon-Ayala

After the publication of this article [1], concerns were raised that the findings of the study cited as Reference 35 were partially misrepresented in the Discussion. Here, the authors provide an amended version of the text to correct the published record.

In the Discussion section, the second paragraph is partially incorrect and should be disregarded. The correct paragraph is:

The current results evidence the advantages of promoting palm oil production within Colombia, and the recent increase in production reflects this as a priority in the national policy. This statement supports the National Federation of Palm Oil Growers (Fedepalma) projections for expanding domestic and international markets [34, 39]. Nevertheless, the increase in palm oil production might lead to deforestation, as the Southeast Asian region has experienced, resulting in adverse environmental and social impacts [36, 37].
